# Chronic spontaneous urticaria is associated with elevated serum zonulin levels

**DOI:** 10.1038/s41598-026-35394-9

**Published:** 2026-01-20

**Authors:** Büşra Solak Esen, Merve Hatun Erkayman

**Affiliations:** 1 Department of Dermatology and Venereology, Atatürk University Faculty of Medicine, Erzurum, Turkey; 2 Department of Dermatology and Venerology, Bağcılar Training and Research Hospital, Istanbul, Turkey

**Keywords:** Chronic spontaneous urticaria, Intestinal permeability, Zonulin, Biomarkers, Diseases, Immunology, Medical research

## Abstract

Autoimmunity is one of the potential mechanisms that has been proposed in the etiopathogenesis of chronic spontaneous urticaria (CSU). High serum zonulin levels are considered a marker for increased intestinal permeability, which has recently become an important research topic in autoimmune diseases. We designed this study to investigate the relationship between CSU and intestinal permeability based on serum zonulin levels. 65 patients with CSU and 65 age- and sex-matched healthy volunteers were included in the study. Serum zonulin levels were measured simultaneously with enzyme-linked immunosorbent assay (ELISA). Disease activity was assessed with the 7-day Urticaria Activity Score (UAS-7). The mean serum zonulin level was significantly higher in patients with chronic spontaneous urticaria (131.7 ± 49.7 pg/ml vs. 82.8 ± 29.4 pg/ml, *p* < 0.001). Patients with chronic spontaneous urticaria who had concomitant inducible urticaria exhibited higher serum zonulin levels compared with those without inducible urticaria (p = 0.014). No significant correlation was found between serum zonulin levels and age, sex, body mass index, disease duration, concomitant angioedema, and UAS-7 score. The study found that patients with CSU had significantly elevated serum zonulin levels compared to healthy controls, supporting the hypothesis that urticaria is associated with increased intestinal permeability.

## Introduction

Chronic urticaria (CU) is defined by repeated episodes of itchy wheals lasting longer than six weeks, either with or without accompanying angioedema^1^. In contrast to chronic inducible urticaria (CindU), chronic spontaneous urticaria (CSU) has no specific external trigger and accounts for 2/3 of all chronic urticaria^2^. With its long-lasting course, which lasts 3–5 years in 60% of patients, chronic urticaria is associated with a considerable impairment of health-related quality of life, which manifests itself in disturbed sleep quality, restrictions in everyday activities and a considerable financial burden^3^.

Although skin mast cells are known to play a major role in pathogenesis, the mechanism of CSU remains largely unknown. Several causes have been suggested for the development of CSU, including autoimmunity, infections, dysregulation of the coagulation cascade, vitamin D insufficiency, and genetic factors^4^. Autoimmunity, which refers to an abnormal immune response in which the body produces antibodies or immune cells targeting its own tissues, is regarded as the most significant of these potential mechanisms, accounting for approximately 50–60% of the cases^4^. In clinical studies, autoimmunity in CSU is commonly defined by the presence of specific autoantibodies (such as immunoglobulin (Ig) G autoantibodies to either IgE or the high-affinity IgE receptor) or by positive results in autologous serum skin tests (ASST)^5^. Impaired intestinal permeability has been proposed as a mechanism underlying the development of autoimmunity^6^. The intestinal mucosa plays a role in regulating the traffic of environmental antigens between the external environment and human body. Disruption of the intestinal barrier makes humans more sensitive to environmental antigens^6^. The translocation of microbiota stimulating the immune system is also regarded as a mechanism of autoimmunity in impaired intestinal permeability^6^. It has been proposed that increased intestinal permeability may facilitate greater exposure to pseudoallergens, which in turn could contribute to the pathogenesis of chronic spontaneous urticaria^7^. Zonulin, an endogenous molecule discovered in 2000, controls tight junctions in the intestinal epithelium^8^. Zonulin is thought to be an indicator of increased intestinal permeability and studies have found an association between increased serum zonulin levels and autoimmune diseases such as celiac disease, multiple sclerosis, type 1 diabetes mellitus^9^. It has also been reported to be increased in allergic disorders, infectious conditions, and various neoplastic processes^9^. Larazotide acetate, a zonulin inhibitor, has been evaluated as a potential treatment for celiac disease, yielding favorable outcomes^10^. Only a few studies with relatively small patient cohorts have examined the relationship between chronic urticaria and serum zonulin levels, and their findings have been inconsistent. The aim of this study is to investigate serum zonulin in patients with CSU in order to gain insight into the relationship between intestinal permeability and CSU.

## Methods

### Settings and design

This cross-sectional, case-control study was conducted between 01.04.2021 and 25.09.2021 at Atatürk University Faculty of Medicine, Department of Dermatology and Venereology (Erzurum, Turkey). The study was approved by the Atatürk University Faculty of Medicine Clinical Studies Ethics Committee (ethics reference number: B.30.2.ATA.0.01.00 meeting no: 02 Decision No: 21, Approval date: 25.03.2021). All procedures were performed in accordance with relevant institutional/national guidelines and regulations and with the ethical standards of the Declaration of Helsinki (1964) and its later amendments. Written informed consent was obtained from all participants.

Sixty-five patients diagnosed with chronic spontaneous urticaria and 65 healthy volunteers, matched for age and sex, were included in the study. All participants were aged between 18 and 65 years and were recruited after obtaining written informed consent. The diagnosis of CSU was made clinically based on the criterion of urticaria +/− angioedema attacks occurring at least twice a week and lasting for at least 6 weeks, without a specific external trigger. CSU patients are treated according to the guidelines set by the European Academy of Allergy and Clinical Immunology (EAACI) and the Global Allergy and Asthma European Network (GA2LEN)^1^. Individuals diagnosed with autoimmune or autoinflammatory diseases, those with a history of alcohol or drug abuse, allergies, clinical findings consistent with irritable bowel syndrome, malignancies, active infections, liver or kidney diseases, thyroid disease psychosis or neurological disorders, coronary heart disease, diabetes mellitus, as well as those using systemic immunosuppressants or systemic steroids, pregnant women, and individuals who had used antibiotics within the last month or probiotics as dietary supplements were excluded from the study. These exclusion criteria were applied to both the patient group and the healthy control group. Patients with a diagnosis of CSU and laboratory abnormalities related to antinuclear antibodies, anti-dsDNA, anti-TPO, anti-thyroglobulin, and celiac antibodies were also excluded. In the healthy control group, these autoantibodies were not routinely tested.

Patient demographics, body mass index (BMI) disease characteristics, total serum IgE levels, and the presence of Helicobacter pylori antigen in stool were recorded. Disease activity was assessed with 7-day urticaria activity score (UAS-7)^11^. Patients with H. *pylori* infections did not receive eradication treatment. In healthy controls, total IgE levels and H. *pylori* status were not routinely assessed.

## Sample collection and analysis

12 h fasting venous blood samples were collected and centrifuged within 2 h at 1000 × g and 2–8 °C for 15 min. The collected sera were stored at -80 °C until the day of analysis. After the separated sera were dissolved once on the day of analysis, the serum samples were brought to room temperature (18–25 °C) and stored for 20 min according to the manufacturer’s instructions of the commercial kit. The levels of human zonulin in serum samples were detected using a sandwich enzyme-linked immunosorbent assay (ELISA) (SinoGeneClon Biotech Co., Ltd., catalogue number: SG-10802, Hangzhou, China). The sensitivity of the kit was 10 pg/ml. The intra-assay coefficient of variation (CV) was less than 8% and the inter-assay CV was less than 10%.

## Statistics

Statistical analysis was performed using Statistical Package for Social Sciences (SPSS Inc., Chicago, Illinois, USA) v21.0. The normality of the continuous data was assessed using histograms and Q-Q plots and the Kolmogrov-Smirnov, Shapiro-Wilk tests. Homogeneity of variances was assessed using the Levene’s test. Normally distributed continuous data are shown as mean ± standard deviation, otherwise they are shown as median (minimum-maximum). Categorical data were presented as frequencies and percentages. When comparing continuous data between the two groups, either the t-test for independent groups or the Mann-Whitney U-test was used. For the comparison of continuous data between the three groups, the Kruskal-Wallis test was used because of the non-normal distribution of data. The Pearson chi-square test was used to compare categorical data. Spearman and Pearson analyses were used to determine the correlation between two continuous variables. All tests were two-sided, and statistical significance was set at *p* < 0.05.

## Results

A total of 130 participants, including 65 CSU patients and 65 age- and sex-matched healthy controls, were included in the study. The case and control groups were identical in terms of age, sex, and similar in BMI (Table 1). 58.5% (*n* = 38) of the patients lived in urban regions, whereas 41.52% (*n* = 27) lived in rural areas. Of the patients, 72.3% (*n* = 47) were non-smokers, 21.5% (*n* = 14) were smokers, and 4 patients were ex-smokers. Median disease duration was 12 months (1.5–240) and mean serum total IgE level was 188,6 ± 311,6 IU/Ml. Thirty-seven (%56.9) patients had concomitant angioedema, while 28 (%43.1) had concomitant CindU. All patients with accompanying CindU described symptomatic dermographism. 10 patients were using omalizumab while 55 of them had been treated with antihistamines. Thirty (46.2%) participants had UAS-7 scores 15 or lower, while 35 (53.8%) participants had UAS-7 scores more than 15. Patients with omalizumab treatment had significantly lower UAS-7 scores (12/0–21) than patients with antihistamines (20/0–42) (*p* = 0.02).

**Table 1:** Characteristics of the study population.

	All participants (130)	Patients (65)	Controls (65)	*p*-value
Age (mean ± SD)	34.7 ± 11.3	34.7 ± 11.4	34.7 ± 11.3	1.00^1^
Sex n (%)				
Male	46 (%35.4)	23 (%35.4)	23 (%35.4)	1.00^2^
Female	84 (%64.6)	42 (%64.6)	42 (%64.6)	
BMI (mg/cm^2^) (mean ± SD)	26.4 ± 4.3	26.4 ± 4,5	26.4 ± 4.2	0.952^1^

SD: standard deviation, BMI: Body mass index, ^1^: independent groups t test, ^2^: chi-square.

**Fig. 1:**
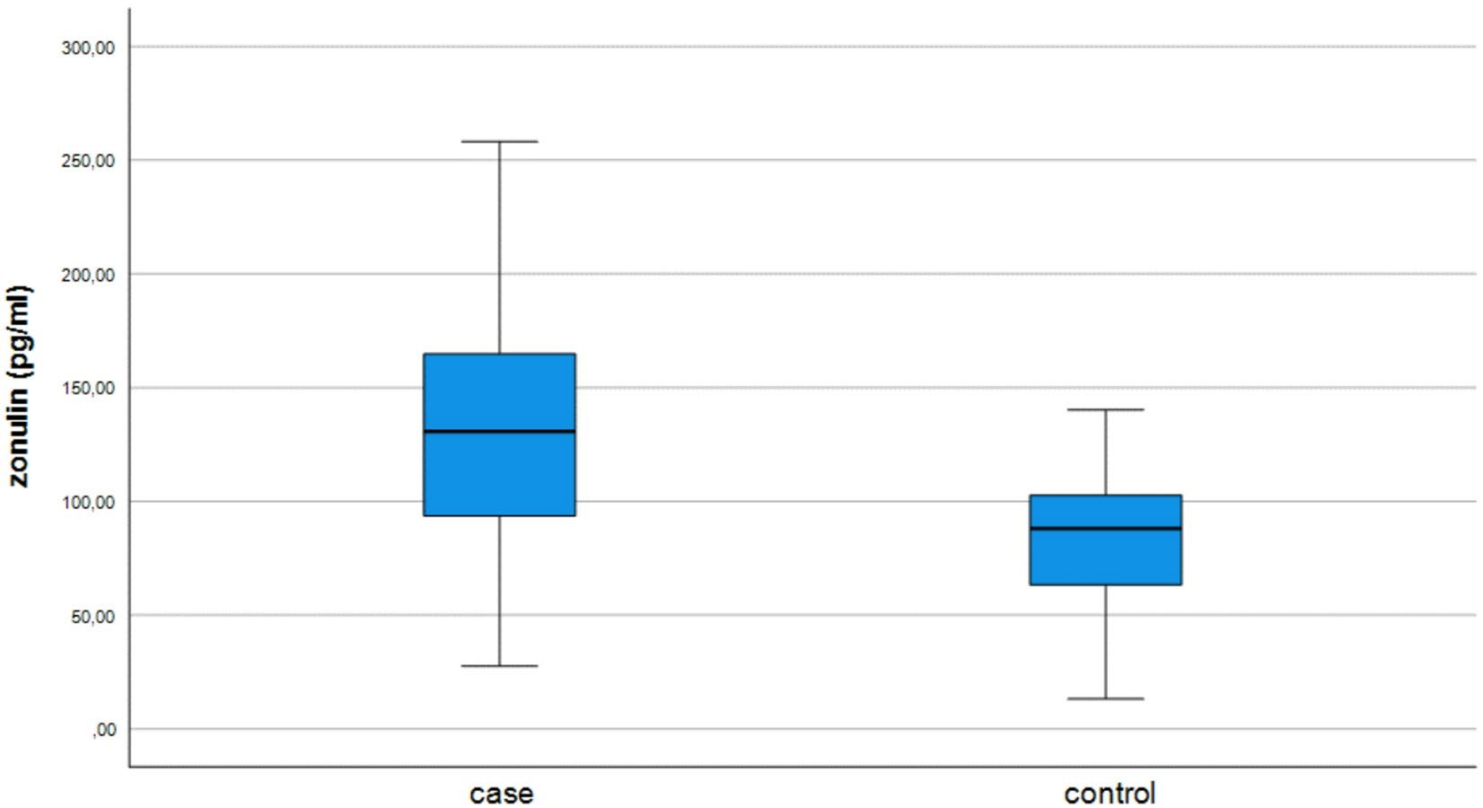
Distribution of serum zonulin levels with 95% confidence interval in patients with chronic spontaneous urticaria (CSU) and healthy controls.

The mean serum zonulin level was 131.7 ± 49.7 pg/ml in the CSU patients and 82.8 ± 29.4 pg/ml in the control group (*p* < 0.001) (Fig. 1). In the study population, no significant association was found between serum zonulin levels and UAS-7 scores, sex, smoking status, place of residence, coexisting angioedema, Helicobacter pylori infection or serum total IgE level (Table 2). The mean serum zonulin level was significantly higher in patients with CindU (148.4 ± 50.9 pg/ml) than in those without CindU (118.9 ± 45.4 pg/ml) (*p* = 0.017) (Table 2). Pearson and Spearman correlation analyses showed no significant correlation between serum zonulin levels and age, BMI, duration of disease or serum total IgE level (Table 3).

**Table 2:** Association between serum Zonulin level and sociodemographic and clinical characteristics in patients with CSU.

		*n* (%)	Serum zonulin (pg/mL) mean ± Std. deviation / median (min-max)	*p*-value
Sex	Male	23 (35.4)	102.6 (73.6–122.0)	0.458^1^
	Female	42 (64.6)	100.9 (77.6–139.0)	
Smoking habit	Yes	14 (21.5)	100.7 (27.5-221.9)	0.166^2^
	No	47 (72.3)	135.8 (58.5-258.2)	
	Ex-smoker	4 (6.2)	130 (94.9-212.3)	
Place of residence	Urban	38 (58.5)	132 ± 49.6	0.947^3^
	Rural	27 (41.5)	131.2 ± 50.8	
Stool *H. pylori* antigen	Positive	7 (10.8)	122 (62.5–159)	0.626^1^
	Negative	58 (89.2)	132.9 (27.5-258.2)	
Angioedema	Yes	37 (56.9)	127.5 ± 54.6	0.274^3^
	No	28 (43.1)	137.3 ± 42.8	
Total IgE (IU/Ml)	0-100	36 (55.3)	125.4 ± 44.8	0.262^3^
	> 100	29 (44.6)	139.4 ± 54.9	
Concomitant CindU	Yes	28 (43.1)	148.4 ± 50.9	**0.017** ^**3**^
	No	37 (56.9)	118.9 45.4	
UAS-7 score *(*n* = 55)	0–15	23 (41.8)	134.6 (27.5-221.9)	0.573^1^
	≥ 16	32 (58.2)	122.5 (54.2-258.1)	

^1^Mann Whitney U test, ^2^ Kruskal Wallis, ^3^independent groups t test.

CindU: chronic inducible urticaria, UAS-7; 7-day urticaria activity score *(Patients treated with omalizumab (*n* = 10) excluded from the analysis).

Significant p values are marked in bold.

**Table 3:** Correlation between serum zonulin levels and age, BMI, disease duration and serum total IgE levels.

	Serum zonulin level	Serum zonulin (pg/mL)
	R	*p*
Age	0.049	0.701^1^
BMI	0.063	0.618^1^
Duration of disease	0.136	0.276^2^
Total IgE	0.010	0.935^2^

BMI: body mass index, ^1^Pearson correlation, ^2^Spearman correlation.

## Discussion

Two different type of autoimmunity developing against self-antigens have been identified in the pathogenesis of CSU: Type 1 (autoallergy), characterized by IgE autoantibodies; and Type 2b, characterized by primarily IgG autoantibodies^12^. The molecular mimicry mechanism of autoimmunity, can occur as a result of increased intestinal permeability. Similar amino acid sequences in foreign and self-peptides lead to the activation of autoreactive B and T cells and subsequently the production of autoantibodies that react with both self and foreign peptides^13^. However the role of intestinal permeability on CSU is less studied. Studies on intestinal permeability in chronic spontaneous urticaria are limited in number and were conducted in small patient groups. The intestinal permeability of 55 patients with CSU was assessed using the sucrose-lactulose/mannitol test, and it was found that the basal gastrointestinal permeability was significantly higher in the patient group than in the healthy controls in a previous study^7^. Zonulin as a marker for intestinal permeability has not been thoroughly investigated in this patient group.

Our study found that serum zonulin levels were significantly higher in patients with CSU than in healthy controls, indicating a link between intestinal permeability and CSU. We also found elevated serum zonulin levels in patients with concomitant CindU, a feature associated with long duration of disease, unresponsiveness to second-generation antihistamines and high disease activity^3,14,15^. Based on this finding we hypothesised that increased serum zonulin levels in concomitant inducible urticaria could be related to increased disease activity in these patients. Ünal et al. have also recently found higher serum zonulin levels in chronic urticaria patients; however the difference was not statistically significant. In the same study, they reported significantly higher serum zonulin levels in patients with higher 7-day urticaria activity scores^16^. In contrast, we found no association between UAS-7 scores and serum zonulin levels. However, our patients were receiving treatment, which is a limitation of our present study. Kamal et al. have found that CSU patients had significant lower zonulin levels than healthy controls^17^. They thought that this situation might be related to the patients’ dietary restrictions or medication use. In another study, Ünal S. et al. found significantly increased serum zonulin levels in CSU patients and showed that serum zonulin levels also increased significantly in the presence of concomitant angioedema^18^. In our study, we did not find any relationship between concomitant angioedema status and serum zonulin level, but we found significantly higher serum zonulin levels in the presence of concomitant CindU. In our study, symptomatic dermographism was detected type in all patients with concomitant CindU. Patients with symptomatic dermographism had higher serum zonulin levels and increased intestinal permeability. We believe that this situation may explain the concepts of food-dependent or food-exacerbated symptomatic dermographism previously mentioned in the literature^19,20^. In these cases, a relationship was found between carbohydrate-rich meal and symptomatic dermographism, and this relationship may be related to the increase in serum zonulin levels caused by carbohydrate-rich meal, especially gluten. Gluten is thought to be one of the major agents that increases serum zonulin levels^9^.

Increased intestinal permeability may represent one of the mechanisms contributing to the pathogenesis of CSU, rather than indicating a direct causal relationship. Mast cell mediators, histamine and tryptase, disrupt the structure of tight junctions and increase intestinal permeability^21^. In previous studies, increased mast cell infiltration was detected in the gastroduodenal mucosa of patients with chronic urticaria^22^. Accordingly, it is possible that mediators released from activated mast cells in the CSU may further increase this permeability. However, it remains unclear whether the elevated serum zonulin levels observed in CSU are predominantly driven by mast cell-derived mediators or initiated by environmental factors. Further studies are needed to elucidate this mechanism.

In the literature, increases in serum zonulin levels are generally associated with increased intestinal permeability. However, it is important to note that increases in serum zonulin levels are not solely attributable to increased intestinal permeability. Current commercial ELISA kits measure not only intestinal-derived zonulin (pre-haptoglobin-2) but also a range of structurally related proteins within the broader zonulin family^23^. Therefore, it may be difficult to interpret high serum zonulin levels as increased intestinal permeability because current ELISA kits need improvement and more specific measurement methods are needed.

In recent studies, serum total IgE level is considered an important marker for determining disease activity, response to treatment and subtypes of CSU. A high total IgE level has been associated with high disease activity, long disease duration, relatively good response to omalizumab and rapid relapse after discontinuation of omalizumab^24^. Very low total IgE levels (< 40) might indicate type t2b autommunity^1^. However, our study did not show an association between serum total IgE and serum zonulin. We thought that serum zonulin levels could be an effective marker in determining autoimmunity subtypes in chronic urticaria, but we could not reach this conclusion due to the limited number of patients.

The inflammatory state caused by Helicobacter pylori infection in the gastrointestinal tract is expected to increase intestinal permeability^25^. Although some studies have reported a higher incidence of H. *pylori* infection in patients with chronic urticaria compared to healthy controls, the role of H. *pylori* as a trigger of chronic urticaria remains controversial. In our study, we failed to show an association between serum zonulin levels and H. *pylori* infection. But the number of patients with H. *pylori* infection was lower (*n* = 7) than expected.

Zonulin as a marker of intestinal permeability has also been studied in patients with other skin diseases. Although previous studies in patients with vitiligo^26^, psoriasis^27^ and rosacea^28^ have shown elevated zonulin levels, it was found to be low in patients with alopecia areata^29^. Overall, these results may support the role of increased intestinal permeability in chronic autoimmune/inflammatory skin conditions. However, further studies are needed to elucidate the complex pathogenesis of these diseases and how exactly increased permeability influences this process.

The present study has several limitations. These include relatively small number of participants, and the ongoing treatment status of the patients, which could potentially alter serum zonulin levels. Additionally, the lack of data on gastrointestinal symptoms represents a limitation that should be addressed in future research.

In conclusion, our study indicates that serum zonulin levels are elevated in patients with CSU and in the subgroup of patients with concomitant CindU. However, due to the study design, it is difficult to make a concrete judgment about the causal relationship. These results may indicate that intestinal permeability is associated with the development of CSU. Future studies could focus on understanding the mechanisms of how intestinal permeability affects certain subtypes of CSU, as well as implementing strategies to regulate intestinal permeability in the treatment of patients with CSU.

## Data Availability

The data analyzed are saved during the current study and available from the corresponding author on request.

## Abbreviations

CU: Chronic urticaria
CSU: Chronic spontaneous urticaria
CindU: Chronic inducible urticaria
